# Priming Maritime Pine Megagametophytes during Somatic Embryogenesis Improved Plant Adaptation to Heat Stress

**DOI:** 10.3390/plants10030446

**Published:** 2021-02-26

**Authors:** María Amparo Pérez-Oliver, Juan Gregorio Haro, Iva Pavlović, Ondřej Novák, Juan Segura, Ester Sales, Isabel Arrillaga

**Affiliations:** 1 Plant Biology Department, Faculty of Pharmacy, Biotechnology and Biomedicine (BiotecMed) Institute, Universidad de Valencia, Vicent Andrés Estellés s/n, Burjassot, 46100 Valencia, Spain; mapeo5@uv.es (M.A.P.-O.); hablas@alumni.uv.es (J.G.H.); juan.segura@uv.es (J.S.); 2 Laboratory of Growth Regulators, Faculty of Science, Institute of Experimental Botany of the Czech Academy of Sciences, Palacký University, Šlechtitelů 27, 783 71 Olomouc, Czech Republic; iva.pavlovic@upol.cz (I.P.); ondrej.novak@upol.cz (O.N.); 3 Laboratory for Chemical Biology, Division of MoLecular Biology, Ruđer Bošković Institute, Bijenička cesta 54, 10000 Zagreb, Croatia; 4 Agrarian and Environmental Sciences Department, Institute of Environmental Sciences (IUCA), University of Zaragoza, High Polytechnic School, Ctra. Cuarte s/n, 22071 Huesca, Spain; esalesc@unizar.es

**Keywords:** heat stress, *HSP*, hormones, *Pinus pinaster*, photosynthesis, priming, ROS, somatic embryogenesis, transgenerational memory, *WRKY*

## Abstract

In the context of global climate change, forest tree research should be addressed to provide genotypes with increased resilience to high temperature events. These improved plants can be obtained by heat priming during somatic embryogenesis (SE), which would produce an epigenetic-mediated transgenerational memory. Thereby, we applied 37 °C or 50 °C to maritime pine (*Pinus pinaster*) megagametophytes and the obtained embryogenic masses went through the subsequent SE phases to produce plants that were further subjected to heat stress conditions. A putative transcription factor *WRKY*11 was upregulated in priming-derived embryonal masses, and also in the regenerated P37 and P50 plants, suggesting its role in establishing an epigenetic memory in this plant species. In vitro-grown P50 plants also showed higher cytokinin content and *SOD* upregulation, which points to a better responsiveness to heat stress. Heat exposure of two-year-old maritime pine plants induced upregulation of *HSP70* in those derived from primed embryogenic masses, that also showed better osmotic adjustment and higher increases in chlorophyll, soluble sugars and starch contents. Moreover, ϕPSII of P50 plants was less affected by heat exposure. Thus, our results suggest that priming at 50 °C at the SE induction phase is a promising strategy to improve heat resilience in maritime pine.

## 1. Introduction

Global warming will challenge forest tree populations in boreal and temperate regions in the next decades. Many species will have to adjust to predicted warmer climates, and conifers may be among the most negatively affected [1]. In the Mediterranean basin, evidence of forest decline during the last decades has already been associated to the combination of high temperatures and droughts, that induce decreased tree growth, massive mortality and die-off events. In this region, mountain forests cope with multiple stresses during summer, such as heat, drought and photoinhibition, and under the on-going climate change scenario, these adverse conditions are expected to become aggravated. This implies that there is an urgent need in developing strategies to increase forest stability against abiotic stresses, therefore, proactive adaptive silviculture is recommended for this region [2].

Breeding forest tree species for abiotic stress tolerance by conventional techniques is complicated, not only because of their long generation time and large size, but also because these traits are regulated by loci that are widespread across the whole genome with both additive and interaction effects [3]. Recently, the stimulation of induced defense/adaptation responses by “priming” has emerged as a promising strategy to increase resilience to adverse conditions. Priming refers to prior exposure to an eliciting factor that makes plants more tolerant to future stress exposure.

Plants can store information from stressful conditions at early embryogenic stages, and then acquire memory to respond more efficiently to future environmental constraints [4,5]. This memory is mediated by epigenetic mechanisms, such as sustained alterations in gene expression, changes in hormonal profiles, and accumulation of signaling proteins and transcription factors [6]. These transcriptional rearrangements induce damage protection, growth regulation, osmotic readjustment, and the coordination of hormonal crosstalk [7].

The priming induced-epigenetic memory involves processes of DNA methylation, histone modifications and small interfering RNA [8]. Phenotypic diversity in arabidopsis has been directly linked to DNA methylation altering transcription [9], while in poplar, DNA methylation shaped drought responses [10]. Yakovlev et al. [11] identified specific noncoding micro RNAs differentially expressed in *Picea abies* that could be involved in epigenetic regulation. Some members of the *WRKY* transcription factors family have been described as priming marker genes associated with chromatin modifications and the methylation and acetylation of histones H3 and H4 [12]. Other studies demonstrated that cold or heat priming can induce cross tolerance [13], since these abiotic stimuli direct or indirectly induced the synthesis of signaling moLecules (second messengers) such as NO, H_2_O_2_, Ca^2+^, and abscisic acid (ABA), which are common in plant responses to cold, heat or drought stresses. These messengers regulate the expression of genes involved in acquiring stress tolerance by mechanisms such as cell membrane fluidity alteration, synthesis of osmoLytes (proline), or enzymes that degrade reactive oxygen species (ROS), such as superoxide dismutase (*SOD*), and ascorbate peroxidase (*APX*), as well as other cellular defenses such as dehydrins and heat shock proteins, and other non-enzymatic components as glutathione, ascorbate, carotenoids, and flavonoids [14].

Heat shock proteins (HSPs) are one of the significant classes of moLecular chaperones that act in response to various stresses, but mainly in response to high temperature. They maintain cellular homeostasis by regulating protein folding and unfolding, in conjunction with their subcellular localization, and eventually the degradation (lysosomal or proteasomal degradation pathway) of unfolded and denatured proteins, either in stressed as well as in unstressed conditions. Studies suggest their role in plant growth and development, particularly in embryogenesis, seed and fruit development. HSPs play a key role in stress-responsive signal transduction, in association with heat stress transcription factors (HSF), for inducing thermotolerance. Particularly, HSPs 70 class regulate the transcription of heat-shock genes, since their interaction with HSFs inhibits trimerization, and thereby binding, of HSFs to heat-shock elements [15].

Previous studies on the adaptation thermotolerance in several forest species demonstrated that photosynthetic activity is one of the vital processes most affected by the increase of temperature [16,17]. In radiata pine thermotolerance acquisition was also related to hormone profile, photosynthetic pigments and carbohydrate contents [18,19].

ABA is the main phytohormone modulating plant response to abiotic stress, since it regulates stomata closure, and activates the production of protective compounds. Although its level is greatly enhanced during responses to stresses associated with dehydration, ABA has, however, an important function in heat stress as well [18,20]. Auxins and cytokinins (CKs), which are crucial regulating plant growth and development, are also involved in stress responses as already reported for radiata pine [18]. Thus, recent research demonstrates that CKs directly participate in stress plant tolerance [21]. Furthermore, many of the interactions between components of the CK signaling pathway and stress responses occur as a result of cross-talk between cytokinin and ABA signaling and metabolism [22], as well as with ethylene and other so-called stress hormones [23]. Some CKs response factors belonging to the AP2/ERF family of transcription factors are emerging as potential integrators of CK and stress responses. In addition, current evidence supports that CKs could be primary receptors in temperature sensing [24].

Taking advantage from the above-mentioned epigenetic memory, it is possible to generate conifer plants pre-adapted to different environmental conditions by introducing priming treatments in somatic embryogenesis (SE) protocols. SE, the inducible process of obtaining multiple embryos from vegetative (somatic) cells, is the most promising vegetative propagation technology for conifers worldwide [25,26], since these species typically lack the capacity to be efficiently propagated using low-cost techniques (e.g., cuttings). SE protocols have been developed by our group [27,28] for maritime pine (*Pinus pinaster* Aiton), a species widely used for afforestation in the North-western Mediterranean area due to its fast growth, and the quality of its timber and oleoresins, as well as to its high phenotypic plasticity and stress tolerance. Furthermore, in Spain large genetic variation in adaptive traits have been found between maritime pine provenances [29,30].

There are several studies dealing with the effects of temperature in *Pinus* spp. SE. For *Pinus pinaster*, we found that increasing temperature from 23 to 28 °C during induction and proliferation of embryogenic cell lines resulted in higher rates of embryo production [27]. A priming treatment at 28 °C during SE of radiata pine also increased differentiation of somatic embryos, which showed altered hormonal profiles [31,32], and finally resulted in plants with different water use efficiency levels when assayed at the greenhouse [33]. Priming using higher temperatures (up to 60 °C) improved somatic embryos production and altered amino acid [34] and CK profiles [35] of embryogenic masses (EMs), while regenerated plants showed differential patterns of gene expression and hormone content [36,37]. Similar experiments were carried out with Aleppo pine [38,39].

Therefore, is likely that maritime pine clonal plants regenerated from somatic embryos that underwent different environmental stresses during their development may display some memory of response to these stresses. Here we present our study regarding heat priming during *Pinus pinaster* SE and heat fitness of SE-derived plants.

## 2. Results

### 2.1. Effect of Priming on Maritime Pine Somatic Embryogenesis

SE response of Pinus pinaster megagametophytes depended on the mother tree (Table 1), being higher in B5 and 1058 (22.1 and 10.9% of explants producing EMs for Soria-Burgos and Galicia provenances, respectively). The percentage of embryogenic explants was higher in megagametophytes from the Soria-Burgos provenance, while a significant reduction in EMs production was observed after priming treatments in megagametophytes from this provenance (Table 1).

Maturation was carried out for a total of 70 proliferating lines (Table 2). Despite the observed decrease in EMs induction in primed explants, mature somatic embryos and germinated plants could be obtained from both provenances and from all the temperature treatments (Table 2). These plants were finally transferred to the greenhouse, where showed similar growth rates. After two years, plants reached 10–15 cm in length, and no phenotypic differences were observed among groups derived from the tested priming treatments. Therefore, these plants were used for abiotic stress experiments.

### 2.2. Effect of Priming on Gene Expression Profile of Embryogenic Lines

**Figure 1:**
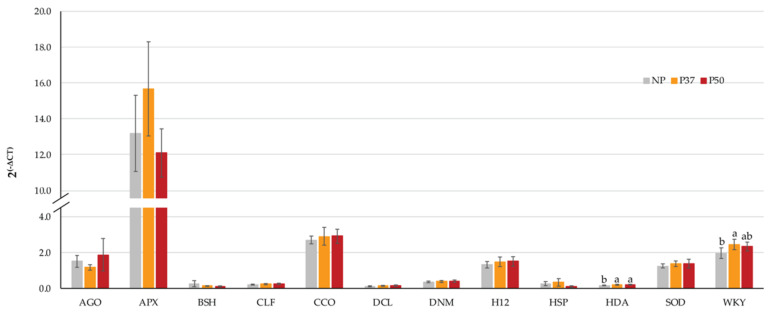
Expression levels of 7 genes regulating epigenetic modifications *(AGO, BSH, CLF, DCL, DNM, H12* and *HDA*) and 5 genes regulating cell response to stress (*APX, CCO, HSP, SOD, WKY*) in maritime pine proliferating embryogenic masses. Data are mean ± SE of four EMs lines (from two mother trees, B5 and B14), that derived from priming at 37 °C × 7 d (P37, orange bars) or at 50 °C × 3 h (P50, red bars), as compared to control EMs induced at 23 °C (NP, grey bars). Gene names are described in Table 3; *TUB* was used as reference gene.

We first examined in primed and control EMs the rates of gene expression for seven genes related to epigenetic modulation that were reported as differentially expressed in *Pinus pinaster* throughout the embryogenesis process [40,41]. Two of them that had been detected at early embryogenesis: *Dicer-like protein* 1 (*DCL*) and *Histone* H1.2 (*H12*), while transcripts of *Argonaute* 9 (*AGO*), *Bushy growth* (*BSH*), *Curly Leaf (CLF*), *DNA (cytosine-5)-methyltransferase 1-like* (*DNM*), and *Histone Deacetylase* 9 *(HDA*), were reported as detected at mid-late embryogenesis. In our analyses, significant differences (*p* = 0.036) among maritime pine EMs were observed only for this *HDA* gene, since expression was higher in primed EMs than in controls (Figure 1). We also investigated the expression of *P. pinaster* genes induced under drought stress [42], a small *Heat Shock Protein70* (*HSP*) and a putative *WRKY*11 transcription factor (*WKY*), and also genes involved in cell response to stress such as those coding for *Ascorbate Peroxidase* (*APX*), *Caffeoyl-CoA O-Methyltransferase* (*CCO*), and a precursor of the *Cu-Zn-superoxide dismutase* (*SOD*). Among them, at this stage, significant differences were observed only for the *WKY* gene (*p* = 0.031), which expression was increased in primed EMs as compared to controls, although difference was not significant for lines from the 50 °C treatment (Figure 1).

### 2.3. Response to Heat Stress of in Vitro Growing Maritime Pine Plants Derived from Primed Embryogenic Lines

Basal expression of *WKY* and *HSP* genes were first determined on in vitro growing plants and then at 3, 24 and 72 h after heat pulses (3 h) were initiated. Basal *WKY* expression was higher in P37 and P50 than in NP plants (n-fold values of 1.38 ± 0.06 and 2.03 ± 0.47 for P37 and P50 plants, respectively). In contrast, basal *HSP* expression was higher in NP than in P37 and P50 plants (n-fold values of 0.88 ± 0.13, and 0.68 ± 0.01, respectively).

**Figure 2:**
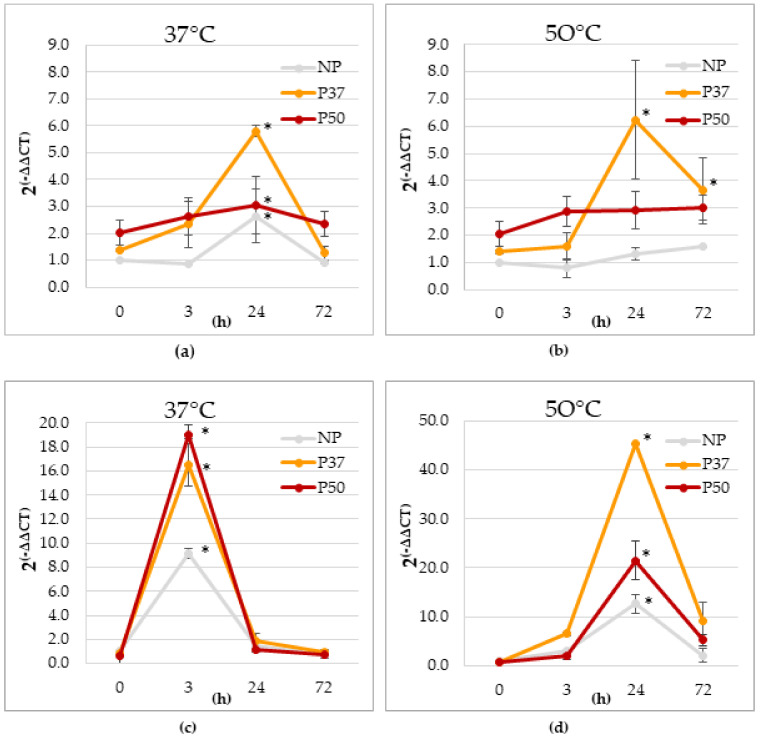
Variation after a 3 h heat pulse of 37 °C (**a**,**c**) or 50 °C (**b**,**d**) in the relative gene expression of *WKY* (**a**,**b**) and *HSP* (**c**,**d**) genes, determined in needles of maritime pine plants growing in vitro. Plants were obtained by somatic embryogenesis, after priming megagametophytes at 37 °C (P37) and 50 °C (P50), as compared to control induction conditions at 23 °C (NP). * significant differences when compared to 0 h data, according to Tukey test (*p* ≤ 0.05).

Heat pulses at 37 °C (Figure 2a) or 50 °C (Figure 2b) for 3 h increased significantly the expression of the *WKY* gene in P37 plants at the end of the stress, but picked (about 6-fold) 24 h after the end of the pulse. Irrespective of the heat pulse (37 or 50 °C), no significant variation was found along with time in the expression of this gene in P50 plants. Expression of *HSP* significantly increased when plants were incubated at 37 °C for 3 h, particularly in primed plants (P37 and P50) and by the end of the heat pulse (Figure 2c). Basal level of *HSP* expression was recovered after 72 h. When the heat pulse was 50 °C for 3 h (Figure 2d), *HSP* expression also increased, being this increment especially notorious in P37 plants. The highest increase relative to the basal expression level of NP plants (up to 40-fold) was observed 24 h after the end of the treatment.

**Figure 3:**
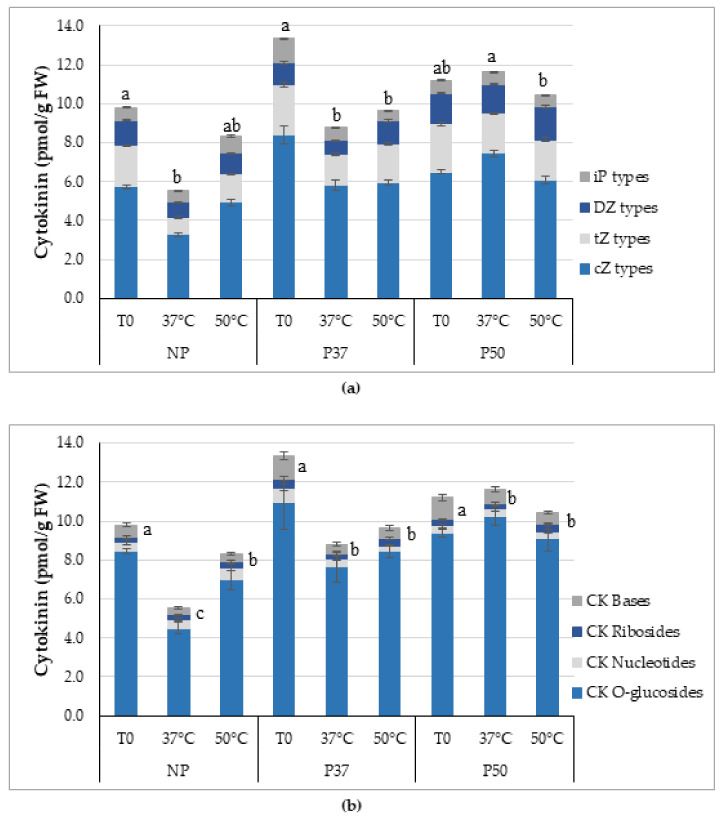
Cytokinin (CK) contents of in vitro growing maritime pine plants derived from control (NP) or primed megagametophytes (P37 and P50), before (T0) and after 3 h of heat stress treatment at 37 °C or 50 °C. Data are mean ± SE of six replicates and 15 compounds grouped by type (**a**) or conjugated structure (**b**). Means separation (Kruskal-Wallis test) for total CK content (**a**) and for CK bases content (**b**) within each group of plants, are also shown. For each provenance, values followed by the same letter were not different according to Kruskal-Wallis test.

We also investigated CKs, ABA and indole acetic acid (IAA) profiles in these in vitro grown plants before (T0) and after a 3 h heat (37 °C or 50 °C) pulse. On average, higher total CKs contents were significantly higher in primed plants than in NP plants (*p* < 0.001), and these differences were detected initially, since basal levels of total CKs were significantly higher in P37 and P50 plants than in NP plants (*p* = 0.001). These significant differences accounted for all the CK types (Figure 3a), and were explained by the higher contents of *c*Z and iP types observed in P37 plants (*p* = 0.001 and *p* = 0.003, respectively), and of total dZ types determined in P50 plants (*p* = 0.002), while levels of total *t*Z types were similar in both groups of primed plants, and significantly higher than those determined in NP plants (*p* = 0.014). After applying a 37 °C heat pulse, total CKs contents were significantly reduced in NP and P37 plants (*p* = 0.001 and *p* = 0.003, respectively), while did not change in P50 plants (*p* = 0.160). The decrease was mainly due to lower levels of *c*Z and *t*Z types (Figure 3a). A pulse at 50 °C did not alter total CKs content of NP and P50 plants, whereas decreased in P37 plants, mainly due to lower levels of *c*Z and iP types (Figure 3a).

On average, levels of active cytokinin bases were significantly lower in NP plants than in P37 and P50 plants (*p* < 0.001), as occurred with CK ribosides (*p* = 0.023), and CK *O*-glucosides (*p* < 0.001), while differences for CK nucleotides contents were significant only for P50 plants (*p* = 0.022). Furthermore (Figure 3b), levels of CK bases were significantly reduced in NP plants after heat pulses (*p* = 0.002 and *p* = 0.017 for 37 and 50 °C treatments, respectively), as occurred for CK *O*-glucosides for the 37 °C pulse (*p* < 0.001), while the other conjugated CKs remained unaffected. When P37 plants were subjected to heat pulses, CK bases, CK nucleotides and CK *O*-glucosides dropped significantly as compared to basal levels (*p* = 0.003, *p* = 0.001, and *p* = 0.003, respectively), while reduction in CK ribosides content was significant only for the 37 °C treatment (*p* = 0.002). For P50 plants, CK bases content was also significantly reduced after heat treatments (*p* = 0.002 and *p* < 0.001, for 37 and 50 °C treatments, respectively). CK ribosides content decreased after a 37 °C pulse, while increased after a 50 °C pulse, therefore we determined significantly different contents in P50 plants after heat treatments. While CK nucleotides in P50 plants did not change after heat pulses (*p* = 0.834), levels of CK *O*-glucosides increased significantly in these plants after a 37 °C treatment (*p* = 0.015).

**Figure 4:**
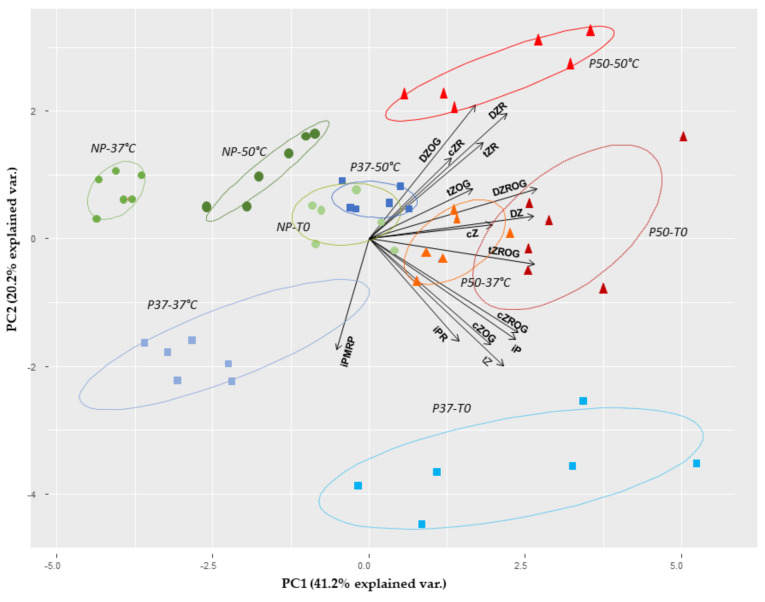
Principal component analysis of 15 cytokinins contents in maritime pine plants derived from control (NP) or primed megagametophytes (P37 and P50), before (T0) and after 3 h of heat stress treatment at 37 °C or 50 °C. Legend: iP, isopentenyladenine; *t*Z, *trans*-zeatin; DZ, dihydrozeatin; iPR, isopentenyladenosine; *t*ZR, *trans*-zeatin riboside; DZR, dihydrozeatin riboside; iPRMP, isopentenyladenosine-5′monophosphate; *t*ZOG, *trans*-zeatin-*O*-glucoside; DZOG, dihydrozeatin-*O*-glucoside; *t*ZROG, *trans*-zeatin riboside-*O*-glucoside; DZROG, dihydrozeatin riboside-*O*-glucoside; *c*Z, *cis*-zeatin; *c*ZR, cis-zeatin riboside; *c*ZOG, cis-zeatin-*O*-glucoside; *c*ZRG, cis-zeatin riboside-*O*-glucoside.

Principal component analysis of the different CKs (Figure 4) clearly separated P50 plants from the other groups and associated CKs ribosides with P50 plants after a heat shock of 50 °C. P50 plants challenged or not with 37 °C were associated to the inactive forms (*O*-glucosides) of the CKs. Finally, iP riboside was associated to P37 plants challenged or not with a 37 °C mild pulse.

**Figure 5:**
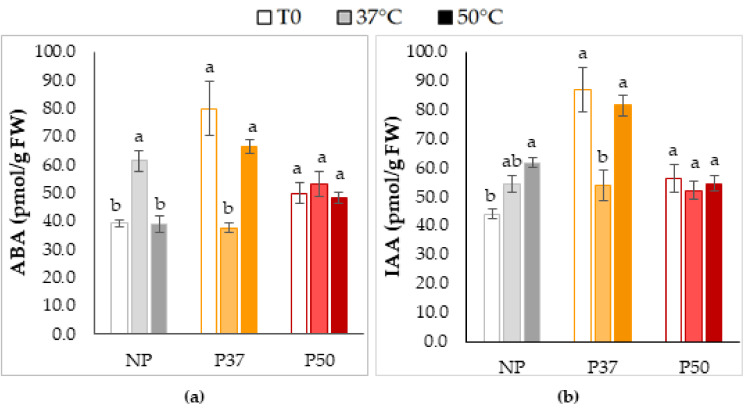
Changes in ABA (**a**) and IAA contents (**b**) before (T0) and after a heat stress treatment (37 or 50 °C for 3 h), in needles of in vitro growing maritime pine plants. Plants were generated by SE from not primed (NP plants, grey bars), or primed at 37 °C (P37, orange bars) or at 50 °C (P50, red bars) megagametophytes. Data are mean ± SE of 4 replicates. For each group of plants, values followed by the same letter were not significantly different according to Tukey (**a**) or Kruskal-Wallis (**b**) tests.

In these in vitro growing maritime pine plants subjected to 3 h heat pulses, we also analyzed ABA and IAA contents, before and after the stress treatment. On average, P37 plants showed higher ABA (Figure 5a) and IAA (Figure 5b) contents than NP and P50 plants (*p* = 0.004 and *p* = 0.005, respectively), but initial hormone contents of these plants decreased after a heat pulse at 37 °C (*p* = 0.005 and *p* = 0.024, respectively), while this treatment significantly (*p* = 0.001) increased ABA level in NP plants. Similarly, IAA content of NP plants significantly increased (Figure 5b) after a heat treatment at 50 °C (*p* = 0.041). In contrast, ABA and IAA contents of P50 plants remained unaffected after either a 37 °C or 50 °C heat treatment.

### 2.4. Response of Primed Maritime Pine Plants to High Temperature Stress in the Greenhouse

Finally, we analyzed whether the primed maritime pine plants were more resilient to heat stress two years after being transferred to the greenhouse. For this purpose, we applied, to these plants, 45 °C for twelve days during 3 h, and measured osmotic adjustment, photosynthetic parameters, carbohydrates content, and expression of genes regulating ROS, before, at the end of the heat treatment, and after a period of recovery to normal conditions.

**Figure 6:**
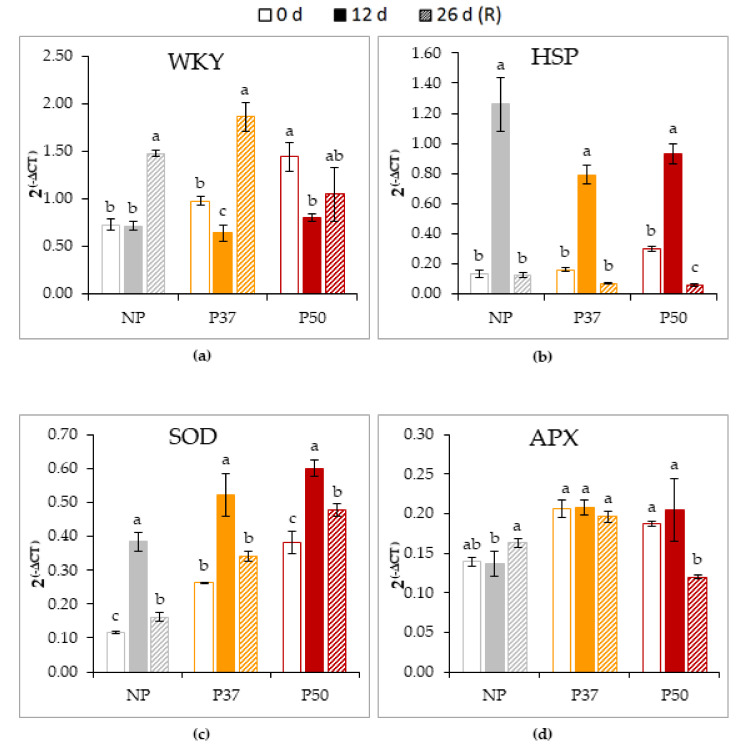
Expression level of *WKY* (**a**), *HSP* (**b**), *SOD* (**c**) and *APX* (**d**) genes in needles of 2-year-old maritime pine plants derived by somatic embryogenesis from not primed (NP) or primed at 37 °C (P37) or at 50 °C (P50) megagametophytes, when subjected to a 12-d heat stress treatment, and allowed to recovery for further 14 d. *HIS* was used as reference gene. Data are mean ± SE of 3 replicates. For each type of sample, values followed by the same letter were not significantly different according to Tukey test.

Gene expression profile showed differences between primed and non-primed plants both before and after the heat stress exposure. Before heat treatment, *WKY* expression was higher in P50 plants than in NP and P37 plants (Figure 6a), while *HSP* expression was similar in NP and in primed plants (Figure 6b). After the heat stress treatment, *WKY* expression was significantly reduced in primed plants, while remained unaffected in NP plants (Figure 6a), and raised drastically after the recovery period in NP and P37 plants. In contrast, heat treatment significantly increased the expression of the *HSP* gene in both primed and non-primed plants, but the increment was greater in NP (Figure 6b). Initial levels of *SOD* expression were also higher in primed plants than in NP plants, and increased significantly after the heat treatment for all the three groups (Figure 6c). Expression levels decreased after recovery, but not enough to resume basal content for NP and P50 plants. Finally, *APX* expression slightly varied in NP plants, while remained unaffected in P37 plants, and decreased only after the recovery period in P50 plants (Figure 6d).

The study of physiological parameters showed that the applied heat stress during 12 d induced differential responses of primed and non-primed plants in osmotic adjustment, photosynthesis-related parameters and total soluble sugars (TSS) and starch content. Parameters were analyzed separately for each group of plants.

#### 2.4.1. Osmotic Adjustment

**Figure 7:**
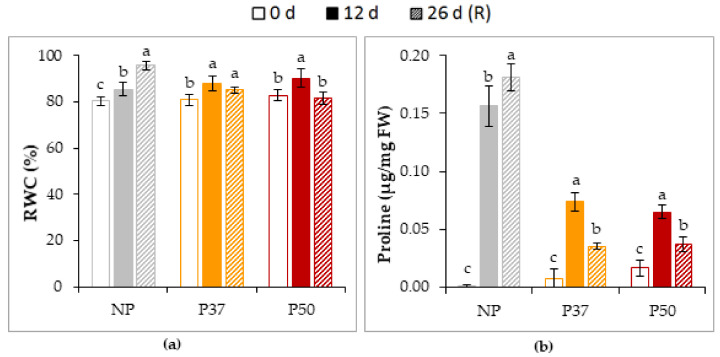
RWC (**a**) and proline content (**b**) in needles of 2-year-old maritime pine plants derived by somatic embryogenesis from not primed (NP) or primed at 37 °C (P37) or at 50 °C (P50) megagametophytes, when subjected to a 12-d heat stress treatment and allowed to recovery for further 14 d. Data are mean ± SE of 3 replicates. For each group of plants, values followed by the same letter were not significantly different according to Tukey test. FW, fresh weight.

Relative water content (RWC) of maritime pine needles significantly increased for the three groups of plants in response to heat stress (Figure 7a), as observed also for proline content (Figure 7b). However, proline content in needles of NP plants doubled values observed in primed plants. After recovery, both RWC and proline content showed even higher values in NP plants, while decreased or remained unchanged in primed plants.

#### 2.4.2. Photosynthetic Parameters

**Figure 8:**
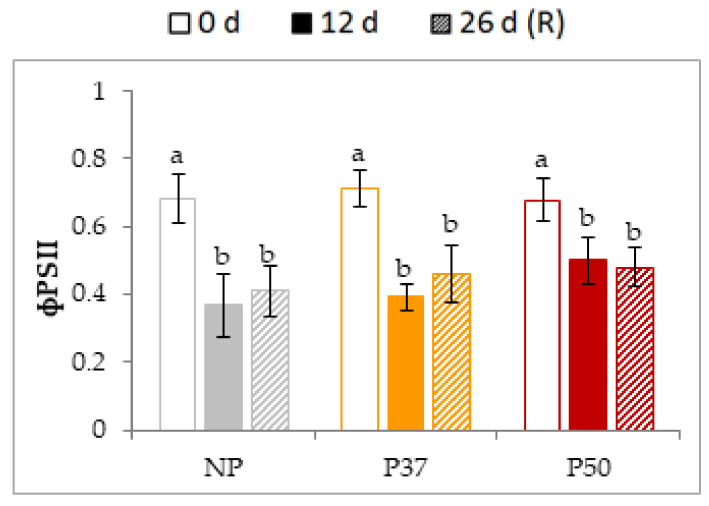
Photosynthetic activity, determined as photosystem II yield, in needles of 2-year-old maritime pine plants derived by somatic embryogenesis from not primed (NP) or primed at 37 °C (P37) or at 50 °C (P50) megagametophytes, when subjected to a 12 d heat stress treatment and allowed to recovery for further 14 d. Data are mean ± SE of 3 replicates. For each group of plants, values followed by the same letter were not significantly different according to Tukey test.

Before the heat treatment (0 d), the Fv/Fm (ΦPSII) yield was about 0.7 with no significant differences among NP, P37 and P50 plants. Heat stress treatment (12 d) decreased photosynthetic yield in all groups of plants (Figure 8) being the P50 (−26%) less affected than NP (−46%) and P37 (−45%) plants. These ΦPSII yields did not significantly increased after the recovery period.

**Figure 9:**
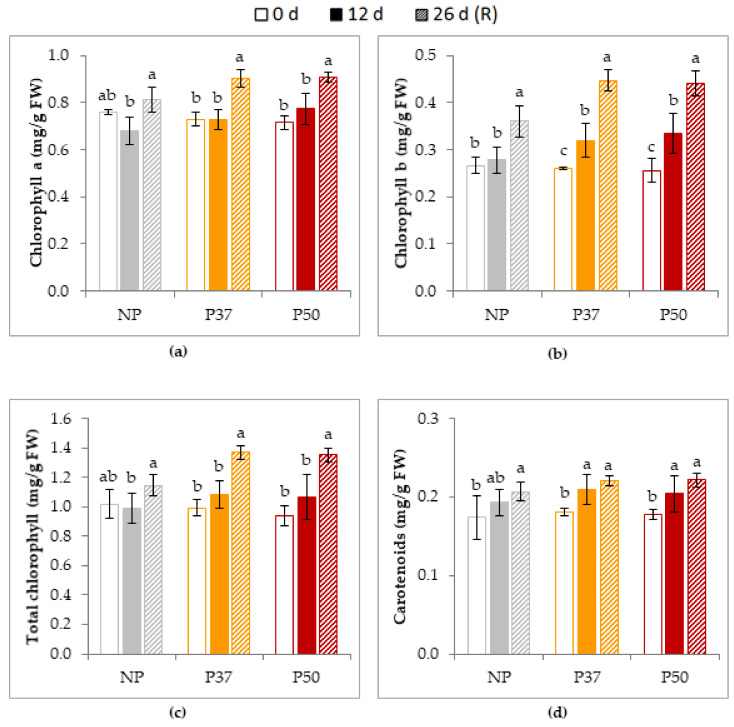
Pigment contents in needles of 2-year-old maritime pine plants derived by somatic embryogenesis from not primed (NP) or primed at 37 °C (P37) or at 50 °C (P50) megagametophytes, when subjected to a 12-d heat stress treatment and allowed to recovery for further 14 d. Data are mean ± SE of 3 replicates analyzed for chlorophyll a (**a**), chlorophyll b (**b**), total chlorophyll (**c**) and carotenoid (**d**) contents. For each group of plants, values followed by the same letter were not significantly different according to Tukey test. FW, fresh weight.

Pigments contents were also analyzed, including chlorophyll a, chlorophyll b, total chlorophyll and carotenoids. Basal contents (0 d) were similar in control and primed plants for the four parameters, and after heat stress remained unaffected chlorophyll a (Figure 9a) and total chlorophyll (Figure 9c) contents, while significantly increased chlorophyll b (Figure 9b) and carotenoids (Figure 9d) contents in primed plants. Mean increase in chlorophyll b for P37 and P50 plants was 27%, and carotenoids increased 15% on average, in contrast to NP plants, which did not show significant variation for these parameters. After the recovery period, chlorophyll a, chlorophyll b and total chlorophylls contents increased significantly (on average 20%, 33% and 23%, respectively) in the three groups of plants, while carotenoids contents did not change, although in NP plants the observed values were significantly higher than those determined after 12 d of heat stress (Figure 9d)

**Figure 10:**
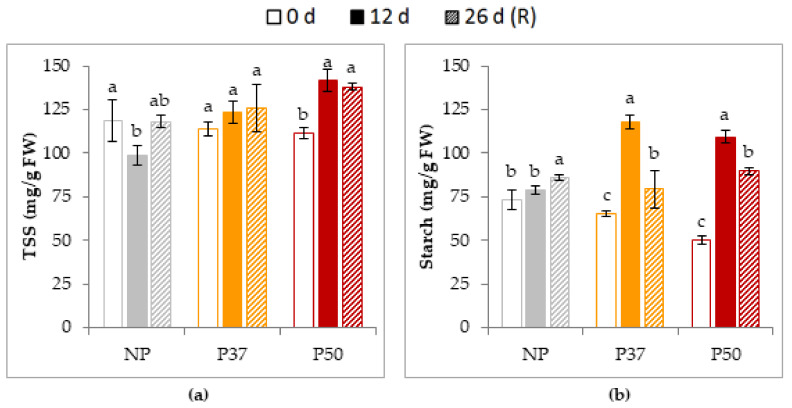
Total soluble sugars, TSS (**a**) and starch (**b**) content in needles of 2-year-old maritime pine plants derived by somatic embryogenesis from not primed (NP) or primed at 37 °C (P37) or at 50 °C (P50) megagametophytes, when subjected to a 12 d heat stress treatment and allowed to recovery for further 14 d. Data are mean ± SE of 3 replicates. For each group of plants, values followed by the same letter were not significantly different according to Tukey test. FW, fresh weight.

Regarding carbohydrate determinations, TSS and starch contents of maritime pine needles were also affected by exposure to heat stress, and varied differentially in primed and in control plants. In NP plants, TSS content decreased significantly (17%) after the heat stress treatment (12 d) and was restored after the recovery period (Figure 10a). In contrast, TSS contents did not varied throughout the experiment in P37 plants while increased significantly in P50 plants (27%). Starch contents were initially lower in primed than in NP plants, and raised significantly after heat stress in primed plants (82% in P37 plants and 118% in P50 plants), while remained unchanged in NP plants (Figure 10b). After the recovery period, starch contents significantly decreased in primed plants (33% in P37 and 18% in P50 plants) while increased (9%) in NP plants.

## 3. Discussion

The main goal of this research was to demonstrate whether priming during maritime pine SE would induce a transgenerational memory that will produce plants with better adaptations to stress conditions. To this end, we applied heat priming to megagametophytes that further underwent through SE process, that is, induction/establishment, maturation, germination and acclimatization [43]. To check for epigenetic changes, we analyzed the expression of several genes related to chromatin modification and adaptation to abiotic stress in EMSs and/or in the regenerated plants after a subsequent heat stress exposure.

The application of heat-priming at the beginning of the SE process negatively affected SE induction but slightly increased (*p* > 0.05) maturation and plant recovery (Table 1). As already reported [27], SE induction was mother tree-dependent; in fact, megagametophytes isolated from genotype B5 produced the highest number of embryogenic lines. The influence of temperature on SE has been reported in several conifer species. Thus, in maritime pine, increasing temperature from 23 to 28 °C during SE resulted in higher embryo yields [27]. In radiata pine, priming at high temperatures (>60 °C), applied at the induction or at maturation stages, produced variable germination responses and alterations in both morphology and stress responses [38].

Histone modification, mediated by methylation, phosphorylation and acetylation, is dynamically regulated during SE and stress responses. Histone methylation seems to be the main mechanism of chromatin regulation in *Picea abies* SE subjected to thermic stress, since other histone modifications (acetylation, phosphorylation, ubiquination and sumoylation) were weakly responsive and therefore not associated to the induction of an epigenetic memory [8]. Because of this, we studied the expression profiles of several genes that in previous studies [40,41] were associated to epigenetic modulation of maritime pine SE. Similar levels of expression were observed in primed and not primed EMs for most of the tested genes, that is: *Dicer like protein* 1 *(DCL*), *histone H1.2 (H12)*, *DNA (cytosine-5)-methyltransferase 1-like (DNMT1)*, *Argonaute* 9 *(AGO*), *Curly leaf (CLF)*, and *Bushy growth (BSH)*. In fact, only the *HDA9* gene was significantly upregulated after priming with middle (37 °C) and high (50 °C) temperatures (Figure 1). Histone deacetylases can contribute to the establishment of epigenetic states and mediate the crosstalk of histone acetylation with other histone modifications [44]. Thus, under drought stress, *HDA9* contributed to repress the expression of the ABA 8′-hydroxylase *CYP707A1* through histone deacetylation, allowing ABA accumulation [45]. In addition, under warm temperature, *HDA9* activates transcription of *YUC8* and thereby auxin biosynthesis [46]. All this may explain the higher IAA and ABA levels found in P37 and P50 plants derived from primed EMs (Figure 5).

Transcription factors (TF) such as those from the WRKY, NAC, DREB and heat sock factors (HSF) families can activate HSP and a battery of genes involved in ROS alleviation [47], thus playing also a pivotal role in priming-derived stress tolerance. Because of this, we investigated in both EMs and its derived plants, the expression of two *P. pinaster* genes previously described as differentially expressed in needles under abiotic stress. Specifically, we tested a putative *WRKY11* TF, a small heat shock protein *HSP70,* and three genes involved in cell response to stress, such as those coding for a precursor of the *Cu-Zn-superoxide dismutase* (*Cu-Zn-SOD*), *ascorbate peroxidase (APX)* and *caffeoyl-CoA O-methyltransferase* (*CCO-MT*). Our results demonstrated that only the putative *WRKY11* gene (*WKY*) was overexpressed in EMs derived from primed megagametophytes, especially those from the 37 °C treatment (Figure 1) indicating that in maritime pine this gene could be of interest as a priming marker. Corroborating this, plants derived from primed megagametophytes maintained the high *WRKY11* expression phenotype while growing either in vitro (Figure 2a,b) and in a greenhouse (Figure 6a).

In vitro growing maritime pine plants were also employed to study the evolution of *WRKY11* and *HSP70* gene expression at several times after short-term heat pulses. *WRKY11* expression did not change immediately (3 h) after a short heat pulse of 37 °C or 50 °C (Figure 2a,b), but significantly increased after 24 h, mainly in P37 plants. Although little research exists regarding *WRKY11* and the response to heat stress, the expression of *WRKY11* in rice, under the promoter of *HSP101*, seems to induce positive responses against heat and water stress [48]. A regulation of *WRKY11* has been also reported in *Arabidopsis thaliana,* where was induced, among other factors, by heat [49]. These evidences, together with our results, allow us to suggest the relationship between heat priming and the increased expression of *WRKY11* that was long- time maintained.

HSPs, also called moLecular chaperones, are highly conserved proteins in plants whose biosynthesis increases in response to high temperatures, playing an essential role in protein stabilization [50]. Several studies associated the expression of a *WRKY11* calmodulin binding transcription factor to heat stress, this TF acting upstream of the HSPs [47] activating *HSFs* that rapidly upregulate expression of various genes, including those involved in the synthesis of HSPs [51]. In our in vitro conditions, a mild heat pulse at 37 °C promptly activated heat responses increasing *HSP70* expression that picked at 3 h (Figure 2c), after which drastically decreased, perhaps due to that the heat shock was too weak to elicit a lasting response. In contrast, when a pulse of 50 °C was applied, the higher expression picked after 24 h, being significantly higher in P37 plants (Figure 2d). This may be caused by some type of internal damage that delays expression, or to the activation of other transcription pathways or factors upstream of HSP70 such as *HSFA1*, a transcription factor that increase the transcription of HSP70 [52,53]. Class 20 HSPs and other small heat shock proteins (sHSPs) seem to play a crucial role in the early thermal response [19,54]. Subsequently, other ATP-dependent HSPs (such as *HSP70*) intervene, binding to the complex and restoring the native conformation of the protein [55]. Thus, we suggest that the synthesis of sHSPs is prioritized during the initial phases of heat shock to avoid proteins aggregation while HSP70s are transcribed in the background and at a slower rate, to act on these aggregates once have been formed. From our results, and irrespective of the heat pulse, it is worth noting the higher *HSP70* expression in P37 plants when compared to P50 at all sampling points.

In arabidopsis, heat stress is associated with alterations in the endogenous levels of CKs and ABA, both seems to be involved in HSP regulation [56]. Particularly, these authors reported that upon exposure to heat stress, ABA content decreased transiently, but levels of active CKs increased in leaves. These changes are associated with the stimulation of transpiration that might help plants to cool down leaf temperature. In our experiments, basal (T0) content of CKs, was higher in P37 than in P50 plants, being always lower in NP (Figure 3a,b). In contrast, the concentration of CK bases in radiata pine was lower in EMs produced after priming at 40 °C in relation to their controls at 23 °C [35]. These differences could be due to the material analyzed, EMs in radiata pine growing in the dark and needles in maritime pine.

The relative abundance of different CKs can vary greatly among plant species, tissues and developmental stages, and depends on the environmental conditions [32]. As previously reported for other pine species [36], the prevailing CKs in *Pinus pinaster* were the *cis*-isomers of Zeatin (*c*Z) and its riboside (*c*ZR), mainly those of the storage CK *O*-glucoside form. Both compounds, *c*Z a *c*ZR, are reported to accumulate under conditions characterized by limited growth and particular developmental stages, but also in response to abiotic and biotic stresses [36,57].

Regarding other hormones, we found very high ABA levels in P37 plants at T0, which suggest a basal protection, since ABA regulates stomata closure as well as production of protective compounds (see [58]). According to a previous report in sage [59], these plants might not need a further increase in ABA under high temperatures. The increased ABA phenotype observed in P37 and, at a less extend, in P50 (Figure 5a) should be attributed to priming during the embryogenesis process. The production of the most active auxin (IAA) was also stimulated by priming (IAA content P37 > P50 > NP plants, Figure 5b). Heat stress (37 °C or 50 °C) increased IAA production in NP plants as described in arabidopsis [20]. Studies of hormonal profiles after heat stress in radiata pine showed that ABA and SA play a crucial role at the first stage of the heat stress response [18]. Nevertheless, in longer exposures and recovery, IAA and CKs seem to be more relevant [19]. Note that heat pulses either at 37 or 50 °C did not altered hormone profiles in P50 plants, probably due to its resilience caused by priming treatment. In this sense, Prerostova et al. [20] reported that acclimation of plants diminished heat shock-induced changes of ABA, JA, CKs, and auxin levels in apices of arabidopsis.

In the experiment performed at the greenhouse, two-year-old maritime pine plants were subjected to heat stress conditions during 12 d. This prolonged treatment significantly increased the expression of the *HSP70* gene (Figure 6b), and also activated *SOD* expression (Figure 6c) in either primed and non-primed plants. In contrast, variation in *APX* expression levels were less evident for the three groups of plants (Figure 6d), and transcription of *WRKY11* only increased after the recovery period (Figure 6a). Among the ROS scavenging mechanisms that reduce the oxidative damage during heat stress [47,60], *SOD* represents the first level of defense against O^2−^ radicals through the formation of H_2_O_2_ and O_2_, while *APX* is one of the enzymes responsible for the conversion of H_2_O_2_ to H_2_O. In our study, the heat stress induced overexpression of *SOD* was higher in primed than in NP plants, therefore these plants could have a better protection from the oxidative damage. This might also explain the observed expression pattern of *APX*, since this gene acts downstream of *SOD* in the defense against ROS. Higher *SOD* expression was also found in a transcriptome study of radiata pine seedlings response after short-term heat stress [19]. Recently, Raja et al. [61] and Sattar et al. [62] showed that SOD and APX enzymes are up-regulated under heat stress in wheat and tomato plants, respectively. In *Citrus*, it has also been seen that an increased antioxidant enzyme is linked to an enhanced ROS scavenging capacity in response to an abiotic stress such as waterlogging [63].

Heat stress is usually followed by a strong decrease in soil water content that leads to the display of symptoms of hydric stress, such as reduction in RWC [42,64]. This would explain the lower RWC determined in needles of NP plants, as compared to P37 and P50 plants, after the application of a 12-d heat treatment (Figure 7a) to maritime pine plants growing at the greenhouse. In *Camellia oleifera,* high temperatures caused greater reduction in the leaf RWC of stressed plants than in to those without stress [65]. When maritime pine plants were allowed to resume control conditions after the heat stress period, RWC of needles from NP plants was significantly increased (Figure 7a), and this recovery of the water losses during the stress period was greater than in P37 and P50 plants, which showed less damage after the application of heat stress.

Osmotic adjustment of plants in response to both biotic and abiotic stresses is also mediated by proline accumulation [66]. In our experiments, proline content was higher (two-fold) in needles of NP than in those from P37 and P50 plants, not only after the heat shock but also after the recovery time (Figure 7b). This suggests that NP plants were more stressed, and therefore needed to accumulate proline to maintain their RWC. However, MoLinari et al. [67] and Borgo et al. [68] demonstrated on sugar cane and tobacco plant, respectively, that high levels of proline in leaves do not contribute to osmotic adjustment under conditions of water deficit, therefore suggesting that stress-induced proline accumulation under water deficit acts as a component of the antioxidant defense system, rather than a mediator of osmotic adjustment.

Thylakoid membranes of chloroplasts are especially sensitive to an increase in fluidity by heat, which can also cause damage to PSII through protein denaturation. Maximum quantum yield of PSII (ΦPSII = Fv/Fm) in native Mediterranean species, including forest trees, decreased to 50% between 41 and 45 °C as compared to 25 °C [16,17,59]; this also holds true in species of agri-food interest such as tomato [69]. Previously, Adams and Demmig-Adams [70] reported that temperature variation (increase or decrease) negatively affects PSII efficiency in conifers, and induced changes in chlorophyll content, which is indicative of an excess of energy causing damage to the photosynthetic apparatus. In our study, ΦPSII values showed a significant decrease in NP and P37 plants after heat exposure, followed by recovery above control values in P37, while NP did not regain the initial values. In contrast, heat stress was less harmful to ΦPSII in P50 (Figure 8). According to this, our results regarding the content of photosynthetic pigments analyzed (chlorophyll a, chlorophyll b and total chlorophyll) showed a smaller decrease during heat stress exposure and a better recovery in primed than in non-primed plants, being the content higher in P50 (Figure 9a–c).

Carotenoid content increased after heat stress in P37 and P50 plants (Figure 9d). Since carotenoids fulfill a double function, as accessory pigments in the capture of light energy, and as moLecules capable of dissipating excess of excitation energy, avoiding PSII damage, this would indicate a better adaptation of plants to heat stress. Similarly, no variations in chlorophyll content during heat stress were reported in radiata pine, but in this species a decrease in this pigment was observed after recovery [18], these authors also reported no carotenoid variation after heat treatments.

Plants response to heat stress induced water deficit that includes, in addition to antioxidant substances that reduce oxidative damage, the accumulation of organic compounds that make the cellular environment more reducing, in an attempt to minimize protein degradation and maintain an energy state as optimal as possible [71]. Soluble sugars, as well as specific levels of glucose, fructose, and mannitol, might accumulate in response to a combination of heat and water stress [72,73,74]. In our experiments, after a 12 d heat stress, primed maritime pine plants showed an increase in TSS content, although this was significant only for P50 plants, while TSS content was significantly reduced in NP plants (Figure 10a). Then, these solutes could play a direct role in the response of maritime pine to high temperature. However, Vinocur and Altman [75] observed that, prior to an increase in the content of TSS, there was a rapid decrease in sucrose, as was also observed in *P. radiata* [18].

Regarding the starch content, we found significant differences among NP, P37 and P50 plants before the heat stress (Figure 10b). However, and despite such differences, after the heat treatment (12 d) primed plants increased significantly their starch content, while this parameter did not change in NP plants. In radiata pine, Escandón et al. [18] did not find differences in starch content between stressed and non-stressed plants. Wang et al. [73] reported that acclimatization of wheat plants to high temperatures effectively improved carbohydrate mobilization and increased starch granules size when plants were subjected to heat stress. Furthermore, starch content variation depended on the type of abiotic stress [76].

From our results, we can conclude that high-temperature priming during the first steps of SE improved adaptation to heat stress in the SE-derived maritime pine plants. These primed plants, especially P50, maintained upregulation of both *WRKY*11 transcription factor and *SOD* genes, that we suggest are responsible of their phenotypes, which are characterized by higher basal levels of hormones (CKs, ABA, IAA) and by differential responses to heat stress, either short-term pulses or prolonged treatments, as compared to NP plants. Under stress conditions, priming-derived 2-year-old plants showed better osmotic adjustment, with a faster recovery of their RWC and a lower increase in proline content, and also a more resilient photosynthetic apparatus, as revealed by lower reduction in ΦPSII yield and in chlorophylls contents. Finally, these plants accumulated higher levels of compounds that contribute to alleviate heat stress- induced damage, such as carotenoids and carbohydrates (TSS and starch).

## 4. Materials and Methods

### 4.1. Plant Material and Production of Primed Plants

Maritime pine cones from six selected mother trees of two Spanish provenances, Galicia (genotypes 1007, 1046, and 1058) and Soria-Burgos (genotypes B5, B14, and B50) were collected in July 2017, and the megagametophytes used as explants for plant propagation by somatic embryogenesis, as described in Cano et al. [43].

Isolated megagametophytes were placed on a modified mLV induction medium [43], and after three days, five petri dishes containing ten megagametophytes each were selected for heat-priming treatments. To this end, petri dishes containing megagametophytes from the Galicia provenance were incubated at 30 °C for 7 d or at 50 °C for 3 h, while those from the Soria-Burgos provenance were primed at 37 °C for 7 d or at 50 °C for 3 h. Then, cultures were transferred to control conditions at 23 °C, and after a total culture period of 8 weeks, the frequency of explants producing embryogenic lines was recorded. Moreover, aliquots (100 mg) of these embryogenic masses (EMs) were placed in microtubes, immersed in liquid nitrogen and stored at −80 °C until analyzing. Maturation was performed as described in Cano et al. [43], and after 12 weeks the number of cotyledonary somatic embryos in each line was determined. After germination, somatic embryo-derived plants were transferred to glass jars for further development. Some of these plants were used for in vitro assays and the remaining were first acclimatized to ex-vitro conditions as described in Arrillaga et al. [27], transferred to the greenhouse, and grown in 2.3 L pots for 2 years, being regularly watered and fertilized. Plants were named, according to the initial megagametophyte-priming treatment, as NP (not primed, control), P37 (primed at 37 °C), and P50 (primed at 50 °C).

### 4.2. Heat Stress Experiments

First, six-months old in vitro growing NP, P37 and P50, plants derived from one embryogenic line (B14 mother tree) were used for testing heat stress response in vitro. Experiments were performed by heat pulses of 3 h either at 37 °C or 50 °C that were applied to 3 glass jars containing 3 plants each, using a factorial design. Needles of these plants were collected before and after 3, 24 and 72 h of the beginning of the heat pulse, and stored at −80 °C for RNA extraction and hormone analyses.

At the greenhouse, the experiment was performed with two-year-old NP, P37 and P50 plants that were subjected to heat stress and allowed to recovery following a modified protocol described in Escandón et al. [18]. Briefly, plants (12 from each group) were first watered up to field capacity, and allowed to stand for 24 h. Heat treatment began with an increasing gradient of temperature from 10 a.m. to 12 a.m., reaching a maximum of 45 °C, which was maintained for 3 h. After that, temperature was gradually decreased from 3 p.m. to 5 p.m. This experimental procedure was repeated for 12 d, and then plants were allowed to recover for 15 d. To avoid collateral drought stress, plants were watered three times per week during the experiment. Mature needles were sampled from plants before (0 d), at the end of heat stress (12 d), and after recovering (27 d), then frozen in liquid nitrogen, and stored at −80 °C until analyzing. Photosynthesis-related parameters were monitored in plants at each sampling time.

### 4.3. Gene Expression Analyses

RNA was isolated from maritime pine frozen samples of EMs growing in proliferating medium for 8 weeks, and from needles of both in vitro and greenhouse-growing plants. RNA from EMs and needles from in vitro-growing plants was isolated using the Plant/Fungi Total RNA Purification Kit (©Norgen Biotek Corp.), while RNA from mature needles of two-year-old plants was isolated following the protocol described by Canales et al. [77]. Genomic DNA was degraded by using the Recombinant DNase I (RNase-free, Takara Bio Inc., Shiga, Japan), following manufacturer’s instructions. RNA quantity and quality were assessed by a NanoDrop^TM^ (Thermo Fisher Scientific, Waltham, MA, USA). Synthesis of cDNA was performed by the PrimeScript^TM^ RT Reagent Kit (Perfect Real Time, Takara Bio Inc.), following manufacturer’s instructions. Real time PCR amplifications were performed in a StepOne Plus (Applied Biosystems, CA, USA), using a final volume of 20 µL containing 0.3 µM of each primer and 10 µL of SYBR Green I Master mix (Takara Bio Inc.) in triplicate for each sample. Amplification conditions were 10 min × 95 °C, and 40 cycles of 15 s × 95 °C and 60 s × 55 °C. Genes coding for *α-tubulin* and *Histone 3* were used as reference to estimate expression levels. Analyzed genes are summarized in Table 3; primer details, designed de novo or described previously by other authors, are given in Supplementary Table S1.

### 4.4. Plant Hormone Analyses

Endogenous contents of abscisic acid (ABA), indoleacetic acid (IAA) and cytokinins (CKs) were determined in *Pinus pinaster* needles sampled from in vitro-growing NP, P37 and P50 plants, before and 3 h after heat treatment. Extraction, purification and quantification of CK metabolites was performed as described by Svačinová et al. [79] using multi-StageTip technology based on C18, SDB-RPS, and Cation-SR sorbents (Affinisep, AttractSPE^TM^, France). Samples were extracted in 1 mL of modified Bieleski solution containing stable isotope-labelled internal standards (0.2 pmoL of CK bases, ribosides and *N*9- and *N*7-glucosides; 0.5 pmoL of CK *O*-glucosides and nucleotides). After extraction, from each sample, three technical replicates of 300 μL were purified using microSPE columns and then eluates were evaporated to dryness.

Concentration levels of IAA and ABA were determined according to modified method described by Šimura et al. [80]. Briefly, samples containing 10 mg fresh weight were extracted in aqueous solution of 50% acetonitrile (*v/v*). Crude extracts were loaded onto conditioned Oasis HLB columns (30 mg/1 mL, Waters) and washed with aqueous solution of 30% acetonitrile (*v/v*). Flow-through fractions containing purified analytes were collected and evaporated to dryness in vacuum evaporator.

All samples were dissolved in 30 μL of mobile phase and then analyzed using an Acquity I-class system (Waters, Milford, MA, USA) combined with a triple quadrupole mass spectrometer (Xevo TQ-S, Waters). A mixture of stable isotope-labelled standards of hormones was added to validate the LC-MS/MS method and concentration levels were calculated using isotope dilution method. All data were processed with MassLynx V4.2 software (Waters).

### 4.5. Characterization of 2-Year-Old Maritime Pine Plants after Heat Stress in Greenhouse Conditions

Relative water content (RWC) was determined according to Escandón et al. [18], using three needle fragments (1 cm long) in each six replicates per sampling point. Samples fresh weight (FW) was registered and needles maintained with de-ionized water for 24 h in dark at 4 °C, after which turgid weight (TW) was recorded. Then, needles were dried at 80 °C for 72 h, and dry weight (DW) was registered. RWC was calculated by using the following equation: RWC (%) = (FW-DW)/(TW-DW) × 100.

Proline was quantified in needles according to Bates et al. [81] with modifications, in six replicates per treatment. About 100 mg of frozen needles were homogenized in 3% sulfosalicylic acid (5 μL/mg FW), and centrifuged at 13,000 rpm for 5 min. A mixture with 100 μL of 3% sulfosalicylic acid, 200 μL of glacial acetic acid, and 200 μL of acidic ninhydrin was added to 100 μL of the supernatant of the extract, and the resulting mixture was vortexed and incubated at 96 °C for 1 h. After that, the reaction was finished on ice for 10 min. Samples were extracted in 1 mL of toluene and vortexed for 20 s, and the formation of two phases was observed. The absorbance of the chromophore-containing toluene phase was read at 520 nm (Eppendorf BioSpectrometer^®^ basic) using toluene as blank reagent, and proline concentration was determined from a Sigma-Aldrich^®^ L-proline standard curve with 6 points (0–150 μg/mL).

To estimate photosynthetic activity in maritime pine plants growing at the greenhouse, (ϕPSII) was analyzed in needles using a pulse-amplitude modulation fluorimeter (MINI PAM; Walz, Effeltrich, Alemania), according to Nebauer et al. [82]. Two measurements of five needles in the mid part of the plant were measured up to a total of ten replicates for each plant group, treatment and sampling period. Needles were pre-adapted in the dark for 20 min and then exposed to a light flash, taking one measure in darkness and another in light, both at a wave length of 350–400 nm. Estimates of ϕPSII were obtained by measuring variable fluorescence (Fv), and calculating the difference (Fv = Fm-F0) between the maximum fluorescence (Fm), after the light flash, and the minimum fluorescence (F0), in the absence of light. The estimated ϕPSII represents the proportion of the energy absorbed by the chlorophyll of PSII that is being used to drive the photochemical process, therefore it is a measure of the efficiency of linear electron transport.

Chlorophyll and carotenoids were extracted and analyzed in needles of maritime pine plants according to Lichtenthaler [83] with some modifications, in nine replicates per treatment. A total of 300 mg of needles were grinded in metal containers with a metal sphere (50 mm ø) using the MM 400-Retsch^TM^ mixer for 30 s. After grinding, three aliquots of 100 mg each were prepared and pigments were extracted in 10 mL of 100% (*v/v*) acetone and centrifugated (10,000 rpm, 10 min, 4 °C). After that, absorbances of the supernatants were read at 470, 645 and 662 nm (Eppendorf BioSpectrometer^®^ basic), and the concentration of each pigment was determined.

Total soluble sugars (TSS) and starch contents were determined in needles of maritime pine plants as described by Rodríguez et al. [84], in six replicates per treatment. Extracts were obtained from 50 mg of frozen needles grinded and homogenized in 10 mL of 80 % (*v/v*) ethanol, by using a MM 400-Retsch^TM^ mixer for 40 s. The mixture was incubated at 80 °C for 1 h and centrifuged (6000 rpm, 20 min, 4 °C). After that, 2.5 mL of a solution of Sigma-Aldrich^®^ anthrone (0.25 g of anthrone in 100 mL of 95% sulfuric acid) were added to 1 mL of the supernatant, and the mixture was vortexed and incubated at 100 °C for 15 min. After cooling down, absorbance was read at 620 nm. Starch content was determined from the pellet resulting from the centrifugation of the initial extract, that was incubated in 10 mL of 30% (*v/v*) perchloric acid for 16 h at room temperature. After centrifugation, 1 mL of the supernatant was mixed with 2.5 mL of anthrone, vortexed and incubated at 100 °C for 15 min. The mixture was cooled down and the absorbance was read at 620 nm. Both TSS and starch contents were calculated against a Sigma-Aldrich^®^ D-glucose standard curve (0–800 μM), using anthrone as a blank.

### 4.6. Statistical Analyses

Data recorded in the different experiments were subjected to analysis of variance using the SPSS software (IBM Statistics). When they did not adjust to a normal distribution (Kolmogorov–Smirnoff test), significant differences were assessed using the Kruskal–Wallis test. PCA was performed using R software [85].

## 5. Conclusions

This work provides new insights for the use of priming during somatic embryogenesis as an important tool in order to induce in plants some memory of response to abiotic stress. In regards to this, the genetic characterization of maritime pine proliferating EMs showed upregulation of *HDA*9 and *WRKY*11 genes in EMs derived from priming at 37 °C and 50 °C, as compared to those derived from not primed megagametophytes. Our results suggested that *WRKY*11 is an important mark for the establishment of epigenetic memory in maritime pine, since its expression was higher in primed than in not primed in vitro growing plants exposed to heat stress. We also demonstrated that this mark is kept in the long term in primed plants at the greenhouse. Furthermore, heat pulses quickly increased the expression of *HSP*70 in primed plants, pointing out this gene, or other factors upstream of *HSP*70, as keys in the early heat response.

Our results also demonstrated that plants derived from megagametophytes primed at 50 °C (P50) were less affected by heat stress. This group of plants showed a higher content of cytokinins than NP and P37 plants, specifically of riboside and riboside-O-glucoside group, and kept stable levels of ABA and IAA during heat pulses. In relation to gene expression, P50 plants showed a higher level of *WRKY* and *SOD* than NP and P37, which suggests a more efficient scavenging of ROS and, consequently, a better responsiveness to heat stress. The physiological characterization of P50 during heat exposure showed a good osmotic adjustment and a higher increase of chlorophyll, TSS and starch content than NP and P37. Moreover, ϕPSII of P50 plants was less affected by heat exposure. Thus, our results suggest that priming is a promising strategy to improve the resilience of maritime pine plants against heat stress, and that the application of a treatment of 50 °C for a short period of 3 h at the induction phase of ES could be the most suitable priming to deal with a subsequent heat stress.

## Figures and Tables

**Table 1:** Effect of mother tree and megagametophyte priming on embryogenesis in maritime pine. Data are mean percentage of explants that produced embryogenic lines after 8 weeks in culture (±SE). (NP) no primed control. NT, no tested.

Provenance	Family	% of Explants with Embryogenic Masses (EMs)				
		Megagametophyte Priming (°C)				
		23 °C (NP)	30 °C	37 °C	50 °C	Mean ^z^
Galicia Mean ^y^	1007	2.5 ± 2.5	3.0 ± 1.5	NT	2.5 ± 1.5	2.0 ± 0.8 b
	1046	9.2 ± 3.2	8.2 ± 3.0	NT	7.3 ± 2.4	6.2 ± 1.3 a
	1058	21.2 ± 3.6	10.2 ± 3.3	NT	12.0 ± 4.7	10.9 ± 2.1 a
		10.9 ± 2.3	7.2 ± 1.6	-	7.1 ± 1.9	
Soria-Burgos Mean ^y^	B5	48.4 ± 3.9	NT	10.1 ± 2.3	29.7 ± 6.4	22.1 ± 3.8 a
	B14	23.9 ± 4.8	NT	0.9 ± 0.9	16.8 ± 4.7	10.4 ± 2.9 b
	B50	18.2 ± 6.2	NT	0.0 ± 0.0	2.4 ± 2.4	5.1 ± 3.0 c
		30.9 ± 3.6 a	-	4.0 ± 1.5 c	18.7 ± 3.7 b	

^z^ Effect of mother tree. For each provenance, values followed by the same letter were not different according to Kruskal-Wallis test; ^y^ Effect of priming. For each provenance, values followed by the same letter were not different according to Kruskal-Wallis test.

**Table 2:** Production of mature somatic embryos and plants from *Pinus pinaster* EMs induced after megagametophyte priming.

Provenance	Priming (°C)	Number of Lines at Maturation	Lines that Produced Mature SE (%)	Number of Recovered Plants
Galicia	23 (NP)	11	72.7	46
	30	13	76.9	99
	50	12	91.7	66
Soria-Burgos	23 (NP)	15	86.7	64
	37	7	100.0	95
	50	12	91.7	125

**Table 3:** *Pinus pinaster* genes analyzed in embryogenic masses and plant needles.

Gene	Gene Annotation	Reference/NCBI Accession
*APX*	*Ascorbate Peroxidase*	AY485994
*CCO*	*Caffeoyl-CoA O-Methyltransferase (CCOMT)*	AM502291.1, JN013969.1
*HSP*	*Heat Shock Protein 70 (HSP70)*	CT577590
*SOD*	*Cu-Zn-superoxide dismutase precursor*	AF434186
*WKY*	*Transcription factor WRKY11*	CT582155
*AGO*	*Argonaute 9 (AGO9)*	de Vega-Bartol et al. [40]
*BSH*	*Bushy Growth (BSH)*	
*CLF*	*Curly Leaf (CLF)*	
*DCL*	*Dicer-Like 1 (DCL1)*	
*HDA*	*Histone Deacetilase 9 (HDA9)*	
*DNM*	*DNA (Cytosine-5)-Methyltransferase 1 (DNMT1)*	Rodrigues et al. [41]
*H12*	*Histone 1.2 (H1.2)*	
*TUB*	*α* *-Tubuline (α-TUB)*	de Vega-Bartol et al. [78]
*HIS*	*Histone 3 (HIS3)*	

## Supplementary Materials

The following are available online at https://www.mdpi.com/2223-7747/10/3/446/s1, Table S1: Genes analyzed in maritime pine embryogenic masses and needles.
