# Supplementary material for: Priming Maritime Pine Megagametophytes during Somatic Embryogenesis Improved Plant Adaptation to Heat Stress
Source: Plants (Basel). 2021 Feb 26;10(3):446. doi: 10.3390/plants10030446 (PMC7996847; doi:10.3390/plants10030446)
Supplement: Supplementary file 1 [file plants-10-00446-s001.pdf]

Supplementary Table 1. Genes analyzed in maritime pine embryogenic masses and needles.

Gene code	Protein	Sequence	Primers		Tm	Product size
			Forward	Reverse		
APX	*Ascorbate peroxidase*	AY485994[x]	GGTCTGGACATCGCAGTTAG	CCACACCAGCCAACTGATAA	62	91
CCO	*Caffeoyl CoA O-methyltransferase*	AM502291.1[x]	GCCGATGAGGGTCAATTTCT	AATGCAAGGGCTGTGCT	62	110
HSP	*Heat shock protein HSP* 70	CT577590[x]	CACCAGCAGTGGGAATATCA	AGGGCATCTGCAATCCTATC	62	90
SOD	*Cu-Zn-superoxide dismutase precursor*	AF434186[x]	CAATGGCTGCATGTCAACAG	CCATCAGAACCCGCAACTAT	62	117
WKY	Putative *WRKY* factor	CT582155[x]	CCCATTATCCTCCACTAACTCC	GCTGAGAGATCGAACCAGTATAG	62	88
AGO	*Argonaute 9* (*AGO9*)	5048[y]	TGCAGATTCTTGTCCATGCTTT	CTGGCTCGCAGCTAAATGC		
BSH	*Bushy Growth* (*BSH*)	7514[y]	GGATTAGCAATGCTCAAAACAC	GGCGAATGGGAATCAAGTTC		
CLF	*Curly Leaf* (*CLF*)	3877[y]	TGAAAAATACTGTGGGTGTTCGA	CAGCAAAGCACGGGCATT		[40]
DCL	*Dicer like protein* 1 (*DCL*1)	28419[y]	TGAACACCACCAAAAGGAATTG	TCTCGTCTTTGCTTGACTTATC		
HDA	*Histone deacetylase* 9 (*HDA*9)	1009[y]	TCTATCGACCACACAAGGCATATC	CAAGTGCTGTGTATCAGGCGTTA		
DNM	*DNA (C-5)-methyltransferase 1-like (DNMT1)*	Pp29536[z]	GGCTGGGTCACACTAATTCAAAG	TGCTTCAGATCCACACTCCTG		[41]
H12	*Histone 1.2 (H1.2)*	Pp46359[z]	CCCGCTTTTGATTTTGTCTGC	CCTCGGCTTCTTTTCCTTTGC		
HIS	*Histone* 3 (HISTO 3)	BX682612[x]	GCTGAGGCTTACCTTGTG	CCAGTTGTATATCCTTAGGCATAA		[78]
TUB	α-*tubulin* (a-TUB)	sp_80862[x]	ATCTGGAGCCGACTGTCA	TGATAAGCTGTTCAGGATGGAA		

[x] Genbank; [y] Sustainpine; [z] Pp transcript
